# Hydrotime Model Parameters Estimate Seed Vigor and Predict Seedling Emergence Performance of *Astragalus sinicus* under Various Environmental Conditions

**DOI:** 10.3390/plants12091876

**Published:** 2023-05-04

**Authors:** Qibo Tao, Dali Chen, Mengjie Bai, Yaqi Zhang, Ruizhen Zhang, Xiaofei Chen, Xiaotong Sun, Tianxiu Niu, Yuting Nie, Shangzhi Zhong, Juan Sun

**Affiliations:** 1 Key Laboratory of National Forestry and Grassland Administration on Grassland Resources and Ecology in the Yellow River Delta, Qingdao Key Laboratory of Specialty Plant Germplasm Innovation and Utilization in Saline Soils of Coastal Beach, College of Grassland Science, Qingdao Agricultural University, Qingdao 266109, China; taoqibo@qau.edu.cn (Q.T.); zhangyaqi020907@163.com (Y.Z.); 17863909472@163.com (T.N.); nyt980505@163.com (Y.N.); zhongsz@qau.edu.cn (S.Z.); 2 State Key Laboratory of Herbage Improvement and Grassland Agro-Ecosystems, Key Laboratory of Grassland Livestock Industry Innovation, Ministry of Agriculture and Rural Affairs, College of Pastoral Agriculture Science and Technology, Lanzhou University, Lanzhou 730020, China; cdl153394@163.com (D.C.); baimj16@lzu.edu.cn (M.B.); 3 College of Animal Science and Technology, Qingdao Agricultural University, Qingdao 266109, China; abcdefg1230829@163.com (R.Z.); sxtong0525@163.com (X.S.)

**Keywords:** *Astragalus sinicus*, base water potential, correlation analysis, hydrotime model parameters, regression analysis, seed germination, seed vigor, seedling emergence

## Abstract

Seed vigor is an important aspect of seed quality. High-vigor seeds show rapid and uniform germination and emerge well, especially under adverse environmental conditions. Here, we determined hydrotime model parameters by incubating seeds at different water potentials (0.0, −0.2, −0.4, −0.6, and −0.8 MPa) in the laboratory, for 12 seed lots of Chinese milk vetch (*Astragalus sinicus*) (CMV), a globally important legume used as forage, green manure, and a rotation crop. Pot experiments were conducted to investigate the seedling emergence performance of 12 CMV seed lots under control, water stress, salinity stress, deep sowing, and cold stress conditions. Meanwhile, the field emergence performance was evaluated on two sowing dates in June and October 2022. Correlation and regression analyses were implemented to explore the relationships between hydrotime model parameters and seedling emergence performance under various environmental conditions. The seed germination percentage did not differ significantly between seed lots when seeds were incubated at 0.0 MPa, whereas it did differ significantly between seed lots at water potentials of −0.2, −0.4, and −0.6 MPa. The emergence percentage, seedling dry weight, and simplified vigor index also differed significantly between the 12 seed lots under various environmental conditions. *Ψ*_b(50)_ showed a significant correlation with germination and emergence performance under various environmental conditions; however, little correlation was observed between *θ*_H_ or *σ*_φb_ and germination and emergence. These results indicate that *Ψ*_b(50)_ can be used to estimate seed vigor and predict seedling emergence performance under diverse environmental conditions for CMV and similar forage legumes. This study will enable seed researchers, plant breeders, and government program directors to target higher seed vigor more effectively for forage legumes.

## 1. Introduction

Chinese milk vetch (*Astragalus sinicus* L.) (CMV) is a globally important forage legume and is especially widely cultivated in East Asia [1,2]. Because of its good adaptability to diverse environments, high quality and yield potential, and excellent palatability, CMV is commonly used as animal feed worldwide and thus plays an important role in developing animal husbandry [3,4,5]. Additionally, the cultivation of CMV improves soil fertility as a result of symbiotic nitrogen fixation, which leads to reduced chemical fertilizer application during crop production [6,7]. Therefore, CMV is an ideal green manure and rotation plant that performs well in environmentally sustainable cropping systems [8,9,10]. Nevertheless, the poor establishment of small-seeded forage legumes is common [11], especially under inappropriate conditions, such as deep sowing, drought, salinity, alkalinity, and cold stress [12], and has become one of the critical constraints encountered by farmers and agricultural companies [13]. Therefore, improving stand establishment in harsh environments has become a major determinant of CMV cultivation and utilization.

The selection of high-quality CMV seeds is an efficient approach to optimizing seedling establishment and thus achieving a better livelihood and environment. Seed vigor is an important aspect of seed quality [14]; it is defined as the sum of the properties of the seed that determine the level of activity and the performance of a seed or seed lot during germination and seedling emergence [15]. Previous research has suggested the presence of a positive linear relationship between seed vigor and seedling emergence in the field [16,17]. Tao et al. found that there was an advantage in using high-vigor CMV seeds to achieve better seedling emergence, whereas low-vigor seeds caused limited seedling emergence [18]. The selection of high-vigor seeds has also been proven to be helpful for seedling emergence in other forage legumes, such as alfalfa (*Medicago sativa* L.) [19], white clover (*Trifolium repens* L.) [20], and birdsfoot trefoil (*Lotus corniculatus* L.) [21]. Thus, more attention should be paid to seed-vigor testing for forage legumes.

The standard germination (SG) experiment, performed according to International Seed Testing Association, is always used to estimate seed quality [22]. Nevertheless, seed vigor describes the comprehensive characteristics of the seeds beyond SG, and seeds from different commercial sources may have similarly high levels of SG in the laboratory; however, under more unpredictable conditions experienced in the field, these same seeds may have strikingly contrasting abilities to establish plants due to differences in their vigor [23]. Consequently, there is an urgent need to develop more precise methods to estimate CMV seed vigor.

There are many ways to test seed vigor, including electrical conductivity [24], DNA replication [25], accelerated aging [26], controlled deterioration [27], and radicle emergence [14,28]. Previously, Tao et al. indicated that an individual count of radicle emergence may have the potential for CMV seed-vigor testing [18]. The methods based on germination characteristics under stress conditions also have the potential for seed-vigor testing. Water potential is a pivotal environmental factor regulating seed germination and emergence [29]. Many studies have suggested that seed germination can be quantified in response to water potential by the hydrotime model [30,31,32]. The hydrotime model was formulated by Gummerson [33] and Bradford [34] and has three parameters that are meaningful from a biological perspective: (1) the constant hydrotime (*θ*_H_), which is the hydrotime (MPa-hours) to germination; (2) the base water potential (*Ψ*_b(g)_), which is the base or threshold water potential (MPa) defined for a specific germination fraction, g (%); and (3) germination uniformity (*σ*_φb_), which is the standard deviation of *Ψ*_b(g)_ [35,36]. The hydrotime model has been shown not only to effectively predict the effect of water potential on progress toward germination but also to provide an understanding of the physiological status of seed lots or seed populations [37,38]. In particular, previous studies have reported the close relationship between hydrotime model parameters and seedling emergence, and thus seed vigor, in many plant species, including alfalfa [39], cotton (*Gossypium hirsutum* L.) [40], rapeseed (*Brassica napus* L.) [41], and sugar beet (*Beat vulgaris* L.) [42]. It has also been reported that seed priming can increase the germination speed under water stress conditions by reducing *θ*_H_ or *Ψ*_b(g)_ [43,44,45,46], which implies that the change in hydrotime model parameters resulting from seed priming may be closely associated with seed vigor and the performance of seeds/seedlings in unfavorable field environments. However, to our knowledge, little literature is available concerning the description of CMV seed germination in response to water potential based on the hydrotime model, and the hypothesis that hydrotime model parameters can be applied to estimate seed vigor and predict the seedling emergence performance of forage legumes, including CMV, has not been validated. Thus, we put forward the following questions in the present research: (1) Does the hydrotime model quantitatively predict seed germination in response to water potential in CMV seed lots with different levels of seed vigor? (2) Can hydrotime model parameters be used as measures to estimate seed vigor and predict CMV seedling emergence performance under diverse environmental conditions? The answers to these questions will provide important parameters for seed-vigor testing and early-warning signs for seed storage.

## 2. Results

### 2.1. Seed Germination in Response to Water Potential

**Figure 1:**
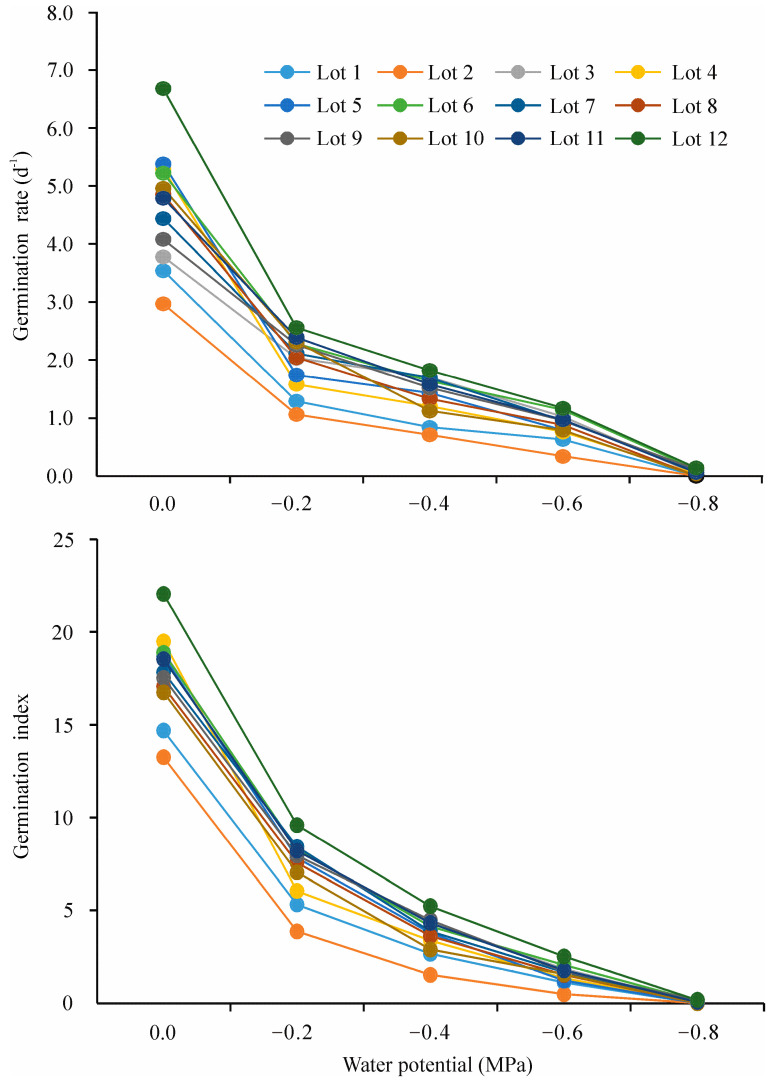
Seed germination rate and germination index of 12 seed lots of Chinese milk vetch (*Astragalus sinicus* L.) in response to water potential. Three replications were performed for each treatment of each seed lot, and each point is the average value of three replications.

The seed germination percentages of 12 seed lots of CMV in response to different water potentials in a laboratory germination test are shown in Table 1. The analysis of variance (ANOVA) indicated that the seed germination percentage (SGP) was significantly influenced by the seed lot, water potential, and their interaction effect (Table 1). Water potential drastically affected seed germination characteristics for both seed lots. With a decrease in water potential, the SGP, germination rate, and germination index decreased significantly. However, the extent of the reduction depended on the seed lot; for instance, from the 0.0 to −0.6 MPa treatments, the SGP of seed lots 2 and 9 decreased from 85.6% to 17.8% and from 90.0% to 51.1%, respectively. When the water potential reached −0.8 MPa, no seed lots germinated to 7.0% (Figure 1, Table 1).

In the non-water stress treatment (0.0 MPa), the germination percentages (standard germination) were high and similar, ranging from 85.6% (lot 2) to 95.6% (lot 12), for the 12 seed lots. In the −0.8 MPa treatment, the SGP also differed little between the 12 seed lots and all lots had values lower than 7%. With the exception of the 0.0 and −0.8 MPa treatments, the SGP differed significantly across the 12 seed lots. For example, in the −0.6 MPa treatment, the germination percentage was significantly affected by the seed lot and ranged from 17.8% (lot 2) to 56.7 (lot 12) (Table 1).

The germination rate and germination index were also significantly influenced by the seed lot in all water potential treatments except for −0.8 MPa, in which low and similar germination rates and germination indexes were observed (Figure 1).

### 2.2. Hydrotime Model Analysis for Seed Germination in Response to Water Potential

**Figure 2:**
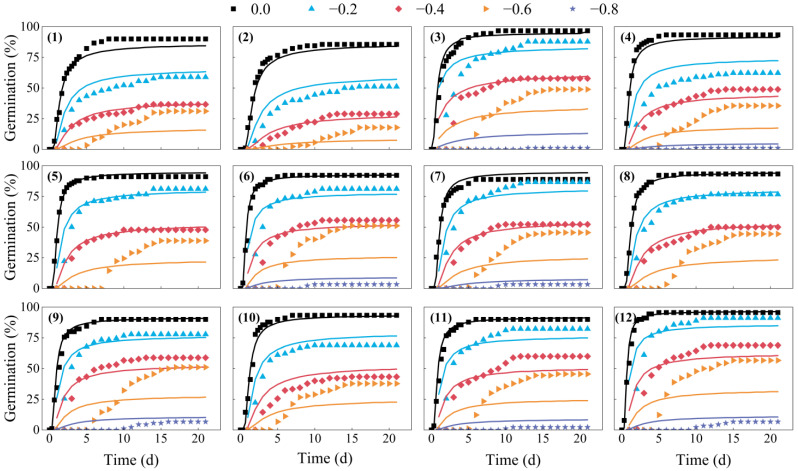
Germination time courses of 12 seed lots of Chinese milk vetch (*Astragalus sinicus* L.). The symbols are the observed data, and the curves are the results of fitting by the hydrotime model. Three replications were performed for each treatment of each seed lot, and each point is the average value of three replications. Numbers in brackets refer to the seed lot.

The hydrotime model parameters calculated from germination data obtained from different water potentials are shown in Table 2. The hydrotime models described the germination of the 12 CMV seed lots well, with *r^2^* values of 0.783 to 0.917 (Table 2). The estimated values of *θ*_H_, *Ψ*_b(50)_, and *σ*_φb_ differed significantly among seed lots. Seed lot 2 had the highest estimated *Ψ*_b(50)_ (−0.278 MPa), and lot 12 had the lowest *Ψ*_b(50)_ (−0.522 MPa). The estimated value of *θ*_H_ of the 12 lots of CMV ranged from 8.799 to 14.770 MPa·h, and *σ*_φb_ varied from 0.267 to 0.333 (Table 2). We also found that the predicted germination time courses at the five water potentials generally fitted the observed germination data very well (Figure 2).

### 2.3. Effects of Different Environmental Conditions on Seedling Emergence Performance in Pot Experiments

The effects of different environmental conditions on the seedling emergence performance of 12 CMV seed lots in pot experiments are reported in Table 3. According to the results of ANOVA, the seed lot and environmental conditions had a significant influence on the pot emergence percentage (PEP), seedling dry weight (SDW), and simplified vigor index (SVI) (*p* < 0.001). Meanwhile, SDW and SVI were significantly affected by the interaction effect between the seed lot and environmental conditions (*p* < 0.001) (Table 3).

**Figure 3:**
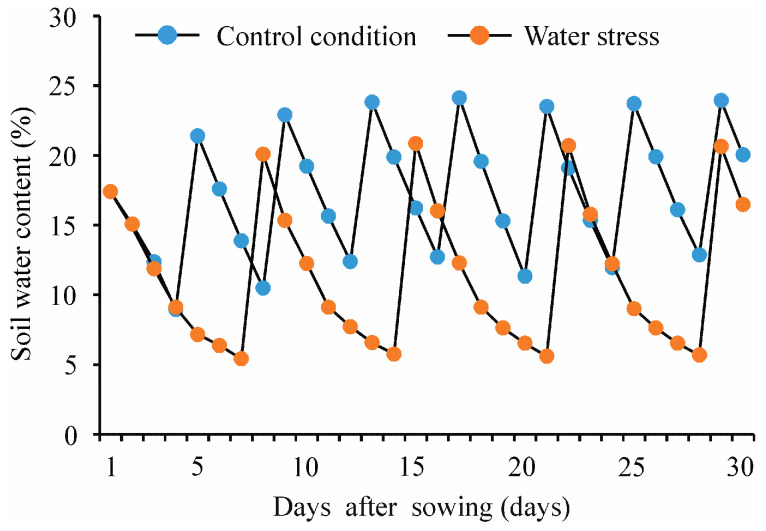
Dynamics of soil water content under control and water stress conditions during pot experiment.

The dynamics of soil water content for control and water stress conditions during the pot experiment are shown in Figure 3. The soil water content was higher in the control condition than in the water stress condition. In the control condition, soil water content in the pot ranged from 8.9% to 24.1% during the pot experiment, with an average value of 17.2%; however, in the water stress condition, soil water content ranged from 5.4% to 20.8%, with an average value of 11.4% (Figure 3).

Overall, PEP, SDW, and SVI decreased significantly with water, salinity, and cold stresses and the deep-sowing treatment as compared to the control conditions and differed significantly across the 12 seed lots of CMV under each environmental condition (Table 3). For instance, for seed lot 6, the PEP under control conditions was 80.0%, while the corresponding values for water stress, salinity stress, deep sowing, and cold stress conditions were 44.4, 46.7, 38.9, and 54.4%, respectively. Among the 12 tested seed lots, the PEP ranged from 54.4 to 83.3%, 28.9 to 52.2%, 26.7 to 53.3%, 15.6 to 43.3%, and 32.2 to 57.8% for control, water stress, salinity stress, deep-sowing, and cold stress conditions, respectively. Generally, seed lot 12 showed the highest pot emergence variables, whereas lot 2 had the lowest (Table 3).

### 2.4. Seedling Emergence Performance under Field Conditions

**Figure 4:**
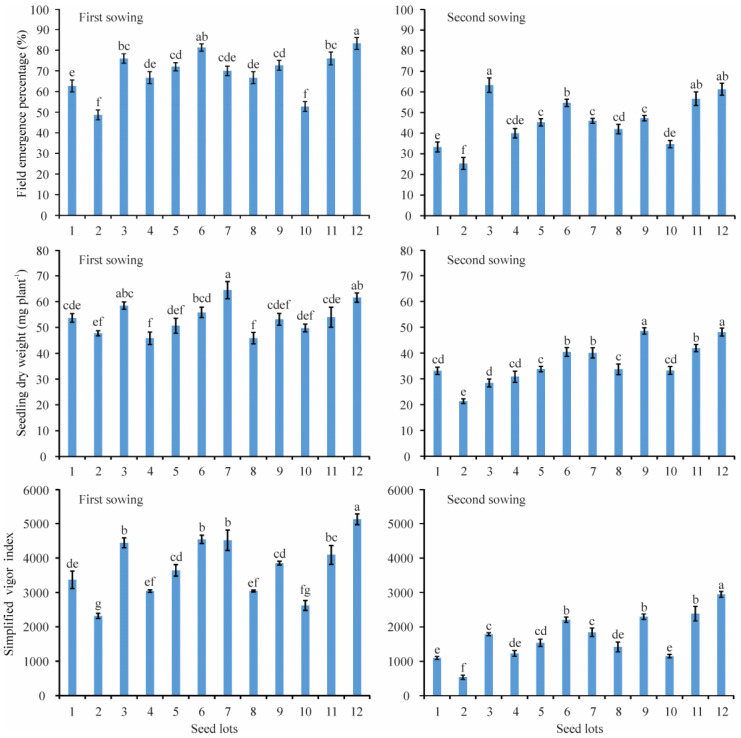
Field emergence percentage, seedling dry weight, and simplified vigor index of 12 seed lots of Chinese milk vetch (*Astragalus sinicus* L.) in two field sowings. Three replications were performed for each seed lot, and each point is the average value of three replications. Different lowercase letters indicate significant differences among different seed lots at the *p* < 0.05 probability level according to Duncan’s multiple comparison test.

There were significant differences in the field emergence percentage (FEP), SDW, and SVI in the two field sowings between the 12 CMV seed lots with similar SG (Figure 4). The observed FEP, SDW, and SVI of all seed lots in the first sowing were higher than those observed in the second sowing. In the first sowing, the FEP, SDW, and SVI for the 12 seed lots ranged from 48.7 (lot 2) to 83.3% (lot 12), 45.8 (lot 4) to 64.4 mg plant^−1^ (lot 7), and 2318.6 (lot 2) to 5123.9 (lot 12), respectively, whereas the corresponding FEP, SDW, and SVI for the 12 seed lots in the second sowing ranged from 25.3 (lot 2) to 63.3% (lot 3), 21.4 (lot 2) to 48.6 mg plant^−1^ (lot 9), and 539.6 (lot 2) to 2946.4 (lot 12), respectively (Figure 4).

### 2.5. Correlation between Hydrotime Model Parameters and Seed Germination and Seedling Emergence Performance under Various Environmental Conditions

**Figure 5:**
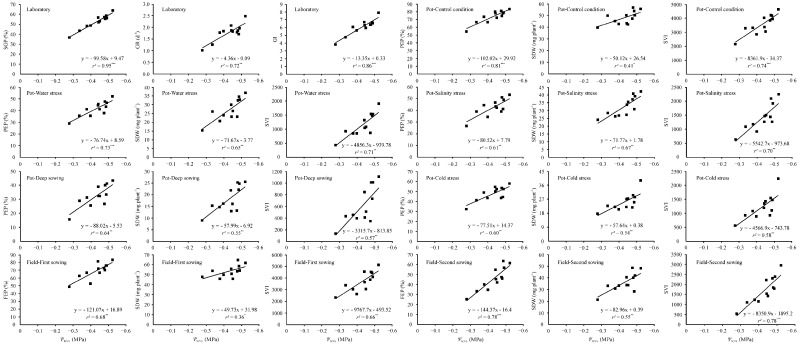
Regression analysis of *Ψ*_b(50)_ with germination characteristics, emergence percentage, seedling dry weight, and simplified vigor index of 12 seed lots of Chinese milk vetch (*Astragalus sinicus* L.) under various environmental conditions. * Indicates significance at *p* < 0.05 probability level, ** indicates significance at *p* < 0.01 probability level, and *** indicates significance at *p* < 0.001 probability level. SGP: seed germination percentage; GR: germination rate; GI: germination index; PEP: pot emergence percentage; SDW: seedling dry weight; SVI: simplified vigor index; FEP: field emergence percentage; *Ψ*_b(50)_: base water potential for 50% of seeds to germinate. SGP, GR, and GI are the overall means with five levels of water potential and three replications. Three replications were performed for each treatment of each seed lot, and the regression analysis was performed using the average value of three replications of emergence data.

Correlation and regression between hydrotime model parameters and seed germination characteristics, emergence percentage, SDW, and SVI under various environmental conditions indicated that *Ψ*_b(50)_ (base water potential) was significantly negatively correlated with the above vigor variables, with the correlation coefficients (*r*) and determination coefficients (*r^2^*) ranging from −0.600 to −0.972 and 0.36 to 0.95, respectively (Table 4, Figure 5). It is evident that seed lots with more negative values of *Ψ*_b(50)_ have higher seed germination and seedling emergence performance under a wide range of environmental conditions. *θ*_H_ (constant hydrotime) was significantly negatively correlated only with the germination rate (*r* = −0.702), germination index (*r* = −0.623), and PEP under cold stress (*r* = −0.630) (*p* < 0.05). In addition, *σ*_φb_ (standard deviation of *Ψ*_b(50)_) was significantly positively associated only with SDW (*r* = 0.666) and SVI (*r* = 0.616) under salinity stress conditions, and SDW (*r* = 0.700) and SVI (*r* = 0.617) in the second sowing under field conditions (*p* < 0.05) (Table 4).

## 3. Discussion

Seed germination and seedling emergence are important stages in the establishment of a plant species and the most sensitive ones in the plant’s life cycle under environmental conditions [47]. In the context of global climate change, drought, salinity, and cold are the major adverse factors limiting seed germination and early establishment as well as subsequent growth and productivity [48,49,50]. In addition, burial depth is also one of the primary factors known to influence seedling emergence and establishment [51], and deep sowing resulted in mechanical stress on seeds and seedlings, which ultimately caused poor emergence performance and stand establishment [52]. As is clearly shown in the present study, reduced water potential significantly decreased SGP, GR, and GI in the laboratory (Figure 1, Table 1), and water stress, salinity stress, deep sowing, and cold stress obviously decreased seedling emergence performance in the pot experiment. We also found that the emergence performance of CMV seed lots was the most sensitive to deep sowing (Table 3). In addition, the seedling emergence percentage under field conditions was far lower than the SG in laboratory (Figure 4), which suggests that the unfavorable environmental conditions experienced in the field play a critical role in determining CMV seedling emergence and stand establishment. These results are consistent with earlier research in other forage legumes and not unusual, such as *Medicago polymorpha*, *Medicago truncatula*, *Medicago littoralis*, *Trifolium alexandrinum*, and *Trifolium michelianum* [53]. In addition, from a seed structure aspect, reduced seed germination and emergence under stressful conditions may also be due to structural changes in the hilum in the seed coat, which is well known to be the main water intake route during legume seed hydration [54]. On the other hand, we found that the extent of the harmful impact of adverse environmental conditions on seed germination and seedling emergence is seed-lot-dependent, which suggests that the selection of high-vigor seeds is a promising method by which to assuage detrimental environmental conditions. Previously, Wang et al. [11] also reported that high-vigor seeds had advantages in the field establishment of the forage legumes alfalfa and purple vetch (*Vicia benghalensis* L.).

In this study, we used 12 CMV seed lots collected from different commercial sources, and our results indicate wide variations in seedling emergence performance among these 12 seed lots under diverse environmental conditions, which effectively rank seed lots according to their actual vigor level. All seed lots used in the present experiment were from a single cultivar; thus, any differences in their germination and emergence patterns and stress tolerance were not due to their genotypes. Therefore, from a seed physiology perspective, the difference in the above traits between seed lots was most likely due to different vigor levels caused by seed aging and deterioration [18]. From the start of seed imbibition to germination or emergence, metabolic repair, including DNA repair, is required and is a necessary step for the subsequent events leading to germination and emergence [55]. Low-vigor seeds germinate more slowly and with more difficulty due to a greater need for damage repair, especially under adverse environmental conditions, which ultimately causes different germination and emergence patterns between seed lots [56]. In our study, among all seed lots, as reflected by seedling emergence under pot and field conditions, seed lot 2 had the lowest vigor level and was also the oldest of all seed lots, stored for six years before its use in the present research. In contrast, the youngest lots (stored for one year: lots 11 and 12) are the high-vigor lots (Figure 1, Table 1 and Table 3). This observation coincides with the above-mentioned aging-repair hypothesis; that is, older seed lots had lower vigor levels. However, the high and similar germination percentages under non-water stress conditions (0.0 MPa) (SG) for 12 commercially available seed lots of CMV were, overall, not strongly associated with seedling emergence performance parameters, including the emergence percentage, SDW, and SVI under different environmental conditions; thus, SG is poor in ranking seed-vigor differences among seed lots. This result is in agreement with the findings of Mavi et al. [57], Yan et al. [58], and Luo et al. [59], who stated that seed lots with high and similar SG may exhibit contrasting performance under stressful conditions experienced in the field. The SG test was carried out under favorable conditions in the laboratory and so may not reflect the actual vigor level of the seed lots [22]. Therefore, more precise testing of seed vigor and quality beyond the SG value is an important area of study for seed researchers [60]. Interestingly, the SGP of the 12 CMV seed lots differed significantly under reduced water potentials, suggesting that the germination percentage under appropriate stressful conditions is beneficial in detecting seed vigor. Our results support those of other researchers who have reported that germination at −0.2 MPa water potential can be used to distinguish the seed vigor of the forage grasses *Festuca sinensis* and *Poa crymophila* seed lots [61,62]. In our study, −0.2, −0.4, and −0.6 MPa were suitable germination conditions for seed-vigor evaluation, since the highest variations among seed lots were obtained at these levels of water potential (Table 1).

The hydrotime model has been widely used to describe germination dynamics in response to reduced water potential [63,64,65], and the model has allowed a better description of seed germination time courses with reduced water availability in many plant species, such as *Stipa* spp. [32], *Ceratonia siliqua* [66], *Jatropha curcas* [67], and slender wheatgrass (*Elymus trachycaulus* (Link) Gould subsp. *trachycaulus*) [68]. Consistent with these experiments, the present study clearly showed that the hydrotime model could also describe the germination time courses of different CMV seed lots at diverse water potentials very well, with *r^2^* values ranging from 0.78 to 0.92 (Figure 2, Table 2). A unique advantage of the hydrotime model for seeds incubated across a range of water potentials is that variation or similarity in germination among seed lots can be ascribed to specific underlying factors, including *θ*_H_, *Ψ*_b(50)_, and *σ*_φb_ [39,69]. In our study, seed lots with lower *Ψ*_b(50)_ generally had higher seed germination and seedling emergence performance under different environmental conditions than those with higher values of *Ψ*_b(50)_; for example, seed lot 12, with the lowest *Ψ*_b(50)_ (−0.522 MPa), also had the highest emergence performance in most cases. In contrast, seed lot 2, with the biggest *Ψ*_b(50)_ (−0.278 MPa), had the poorest seedling emergence under a wide range of environmental conditions (Table 2 and Table 3). This result parallels the findings of Dahal and Bradford [70], who observed that seeds with lower *Ψ*_b(g)_ generally had higher vigor than those with high values, and this allowed seeds to germinate rapidly in adverse environments. Furthermore, our study revealed very significant correlations between *Ψ*_b(50)_ and seed germination characteristics and seedling emergence performance under diverse conditions (Figure 5, Table 4), which supports our hypothesis that the tolerance of CMV seed lots to unfavorable environmental conditions can be identified by a reduction in *Ψ*_b(50)_. Therefore, *Ψ*_b(50)_ could be considered an ideal indicator when determining seed vigor and predicting emergence performance under various conditions. Accordingly, in the present study, the close association of *Ψ*_b(50)_ with seed vigor and seedling emergence performance is in agreement with the previous findings of Chen et al. [39] in alfalfa, Soltani and Farzaneh [40] in cotton, Soltani et al. [41] in rapeseed, and Bradford and Somasco [71] in lettuce (*Lactuca sativa* L.). Farzane and Soltani [42] also suggested that *Ψ*_b(50)_ is significantly negatively related to the seed vigor of sugar beet.

However, in the present experiment, a low correlation was observed between *θ*_H_ and *σ*_φb_ and seed vigor. One probable reason is that a lower *θ*_H_ favors rapid and uniform germination, and this happens only when seeds are exposed to favorable conditions. According to the hydrotime definition, when the external water potential approaches or is lower than *Ψ*_b(50)_, seeds will accumulate hydrotime units very slowly, which overrides the advantage of low *θ*_H_ [39]. In addition, no relationship between *Ψ*_b(50)_ and *θ*_H_ was observed, which implies that these two parameters may act independently in regulating seed germination and seedling emergence under diverse conditions. Accordingly, Farzane and Soltani also indicated that *Ψ*_b(50)_ was the only parameter that was significantly related to seedling emergence and seed vigor [42].

## 4. Materials and Methods

### 4.1. Seed Materials

Samples of 12 seed lots of CMV were obtained from various commercial sources in China. For each lot, sufficient seeds were collected (in excess of 1000 g) to ensure the implementation of subsequent experiments. These seed lots had been produced over several years (Table 5) from diverse regions in China and stored dry at 4 °C before they were used in the present study. The basic information, including the initial seed moisture content, thousand-seed weight, and proportion of hard seeds in the 12 seed lots (ranging from 0.0 to 4.4%), is also presented in Table 5. The experiments described here were conducted from May to December 2022.

### 4.2. Germination Test

In order to calculate hydrotime model parameters for each seed lot, a germination test for each CMV seed lot was carried out by incubating seeds at 20 °C in an 8 h/16 h light/dark cycle at water potentials of 0.0, −0.2, −0.4, −0.6, and −0.8 MPa. Water potentials were maintained with solutions of polyethylene glycol 6000 (PEG-6000) prepared according to Michel and Kaufmann [72]. For each seed lot and treatment, three replications of 30 seeds were used, and seeds were sown in 120 mm × 120 mm Petri dishes on the top of two layers of filter paper moistened with 13 mL PEG-6000 solutions or distilled water; the Petri dishes were then sealed with parafilm to reduce the speed of evaporation of water. Seeds were transferred to new filter paper with fresh solution every 48 h to ensure relatively constant water potential. Germination (2 mm radicle emergence) was monitored every eight hours for the first 4 days from the beginning of the germination experiment, and then daily for 21 days. The germinated seeds were removed at each counting. The germination percentage was expressed as germinated seeds divided by total sown seeds. The germination rate (d^−1^) was calculated as previously described by Fallahi et al. [73]:

$$GR=\sum_{i=1}^{n}\frac{Si}{Di}$$

where GR is the germination rate (d^−1^), *Si* is daily seed germination, *Di* is the number of days to *n* computations, and *n* is the number of computation days. The germination index was calculated using the following formula [74]:

$$GI=\sum \frac{Gt}{Dt}$$

where GI is the germination index, *Gt* represents the number of germinated seeds on day *t*, and *Dt* represents germination days.

### 4.3. Pot Experiments

Cylindrical plastic pots (inner diameter, 14 cm; height, 12 cm) were used in this part of the experiment. The clay loam soil (silt 34%, clay 37%, sand 29%, pH 7.20, and organic matter 17.39 g kg^−1^) used in pot experiments was collected from local farmland and sieved through a 2 mm sieve to remove debris. The soil was dried at 120 °C for 24 h and then spread out for drying, followed by heating and homogenization. CMV seeds in 12 seed lots were sown in various environmental conditions, including control (irrigated with distilled water every four days), water stress (irrigated with distilled water every seven days), salinity stress (irrigated with 125 mM NaCl solution every four days), deep sowing (physical stress) (5 cm), and cold stress (10 °C). The abiotic treatments for the control, water stress, salinity stress, and deep sowing were conducted in a greenhouse (20 °C, 60% relative humidity) on the campus of Qingdao Agricultural University (36°19′ N, 120°23′ E, 38.6 m above sea level), Shandong Province, China. The cold stress treatment was performed in a growth chamber. For each seed lot and treatment, three replicates of 30 seeds were sown at a depth of 1 cm (except for the deep-sowing treatment) in pots that contained 950 g of dry soil, which had been treated as described above. After sowing, 200 mL of distilled water or NaCl solution was used to irrigate each pot immediately and every four days during the period of the pot experiment (except for the water stress treatment, in which pots were irrigated every seven days). The pots containing soil and seeds were placed in a random arrangement on a table in the greenhouse or in the growth chamber, and the locations of pots were changed daily to avoid edge effects.

To monitor the soil water content for the control and water stress treatments, two extra pots without seeds were irrigated under the same conditions as those in each irrigation regime of the experiment. These two pots were weighed daily using an electronic balance, and the soil water content was calculated according to the following formula [75]:

$$SWC\left(\%\right)=\frac{Ww-950}{Ww}\times 100$$

where SWC is the soil water content (%), and *Ww* is the weight (g) of wet soil weighed every day.

The pot experiments lasted 30 days. At the end of the experiment, the number of emerged seedlings was counted in each pot, and the emergence percentage was calculated. All emerged seedlings in each pot were cut at the soil level and combined before being dried at 65 °C for 48 h and then weighed; the mean SDW was then calculated. The SVI was derived arithmetically by multiplying the emergence percentage with the SDW [76].

### 4.4. Field Experiments

The field emergence experiments were implemented at the Jiaozhou Experimental Base of Qingdao Agricultural University (36°25′ N, 120°4′ E, 27.0 m above sea level), Jiaozhou, Shandong Province, China. The mean annual temperature and mean annual precipitation were 13.8 °C and 686.0 mm, respectively. The field emergence experiment was conducted twice, once in June 2022 and once in October 2022. During each sowing, a randomized complete block design (RCBD) with three replications was used, and each replication contained 50 seeds. The seeds from each seed lot and each replication were hand-planted in a 1.5 m row, with 50 cm spaces between adjacent rows (seed lots). The seeds were sown at a depth of approximately 1 cm.

**Figure 6:**
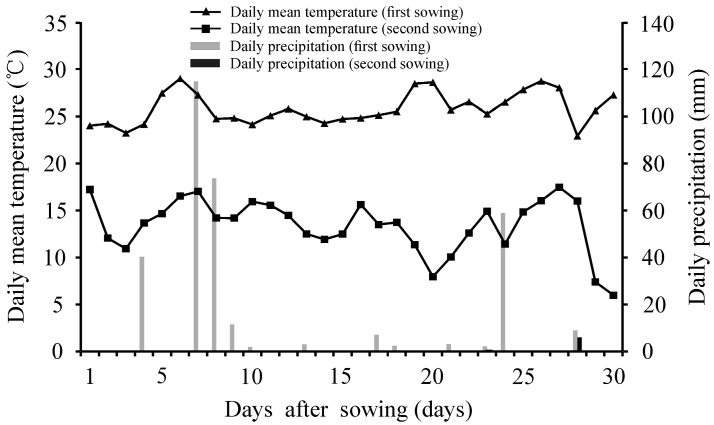
Daily mean temperature and precipitation during two field emergence experimental periods in Jiaozhou, Shandong Province, China.

The daily mean temperature and daily precipitation data during each experimental period were collected from local meteorological stations (Figure 6); no irrigation was applied during the field trials. For the first sowing, the mean temperature and total precipitation during the field trial period were 25.8 °C and 327.9 mm, respectively, while the counterpart values for the second sowing were 13.4 °C and 6.2 mm, respectively (Figure 6). At 30 days after each sowing, the emerged seedlings were counted, and the emergence percentage was calculated.

After each field experiment, all emerged seedlings from each block and each seed lot were harvested at the ground level and amalgamated before being dried at 65 °C for 48 h and then weighed, and the mean SDW was calculated; the SVI was also calculated as described above.

### 4.5. Statistical Analyses

All statistical analyses were performed using SPSS 26.0 software (SPSS Inc., Chicago, IL, USA). Data for germination and emergence percentages in response to the seed lot and water potential were subjected to ANOVA. Significant differences between treatments or seed lots were compared using Duncan’s multiple comparison test at the *p* < 0.05 probability level. Proportional data were arcsine-transformed before statistical analysis, and non-transformed data are reported in all tables and figures. All measurements reported are the mean of three replications. Pearson correlation and regression analyses were used to detect the relationships between hydrotime model parameters and the germination percentage, germination rate, germination index, and seedling emergence performance under various environmental conditions.

The hydrotime model is described by the following equation [33,34,77]:

*θ*_H_ = (*Ψ* − *Ψ*_b(g)_) tg

where *Ψ* is the actual water potential (MPa), *θ*_H_ is the hydrotime constant (MPa·h), *Ψ*_b(g)_ is the base water potential (MPa) defined for a specific germination fraction (*g*), and tg is the time (h) to germination of fraction *g* (%) of the seed lot.

The normal distribution of *Ψ*_b(g)_ values among seeds in a population is characterized by its median *Ψ*_b(50)_ and standard deviation (*σ*_φb_), which can be estimated using repeated probit analyses, varying *θ*_H_ until the best fit is reached [40,69,78] as follows:

Probit (g) = [*Ψ* − (*θ*_H_/tg) − *Ψ*_b(50)_]/*σ*_φb_

which separately models the germination time course at different water potentials for each seed lot.

## 5. Conclusions

Overall, the present study clearly shows that the hydrotime model can describe the germination time course of CMV seed lots in response to different water potentials very well. The 12 seed lots of CMV differed significantly in their hydrotime model parameters (*θ*_H_, *Ψ*_b(50)_, and *σ*_φb_). Additionally, different water potentials and environmental conditions also had a significant influence on seed germination and seedling emergence for each CMV seed lot. Seed germination and emergence characteristics significantly decreased under reduced water potential and abiotic stresses. In addition, the 12 seed lots also significantly differed in their seedling emergence under field conditions. Among the three hydrotime model parameters, correlation and regression analyses indicated that *Ψ*_b(50)_ was the most highly correlated with germination characteristics and seedling emergence performance under different environmental conditions. The lower the *Ψ*_b(50)_ of a lot, the higher its vigor. Thus, it can be concluded that *Ψ*_b(50)_ is an ideal trait to estimate CMV seed vigor and predict seedling emergence under diverse environmental conditions. These findings provide important parameters for seed vigor testing and early-warning signs for CMV seed storage. Future seed treatment and breeding programs for seed vigor could pay attention to reducing *Ψ*_b(50)_ to increase seed vigor and stress tolerance. Moreover, the evaluation of the relationship between stress tolerance characteristics and *Ψ*_b(50)_ might reveal similar significant associations in other plants.

## Figures and Tables

**Table 1:** Seed germination percentage (%) of 12 seed lots of Chinese milk vetch (*Astragalus sinicus* L.) in response to water potential.

Seed Lot	Water Potential (MPa)				
	0.0	−0.2	−0.4	−0.6	−0.8
1	90.0 abc	58.9 ef	36.7 cd	31.1 de	0.0 c
2	85.6 c	51.1 f	28.9 d	17.8 e	0.0 c
3	96.7 a	87.8 ab	57.8 ab	48.9 abc	1.1 c
4	93.3 abc	62.2 ef	48.9 bc	35.6 cd	1.1 c
5	91.1 abc	81.1 bc	47.8 bcd	38.9 bcd	0.0 c
6	92.2 abc	81.1 bc	55.6 abc	51.1 abc	3.3 abc
7	88.9 bc	86.7 abc	52.2 abc	45.6 abcd	3.3 ab
8	93.3 ab	76.7 cd	50.0 bc	44.4 abcd	0.0 c
9	90.0 abc	77.8 cd	58.9 ab	51.1 ab	6.7 a
10	93.3 abc	68.9 de	43.3 bcd	37.8 bcd	0.0 c
11	90.0 abc	82.2 bc	60.0 ab	45.6 abcd	2.2 c
12	95.6 ab	91.1 a	68.9 a	56.7 a	6.7 ab
Analysis of variance					
Source of variance	Degrees of freedom	Sum of squares	Mean square	*F*	*p*
Seed lot (*SL*)	11	1.582	0.144	15.653	<0.001
Water potential (*WP*)	4	28.077	7.019	764.101	<0.001
*SL* × *WP*	44	0.892	0.020	2.206	<0.001

Different lowercase letters within a column indicate significant differences at the *p* < 0.05 probability level according to Duncan’s multiple comparison test. Germination percentage data were arcsine-transformed before statistical analysis, and non-transformed data are shown in the table. Three replications were performed for each treatment of each seed lot, and each point is the average value of three replications.

**Table 2:** Estimated hydrotime model parameters for 12 seed lots of Chinese milk vetch (*Astragalus sinicus* L.).

Seed Lot	*θ*_H_ (MPa·h)	*Ψ*_b(50)_ (MPa)	*σ*_φb_	*r* ^2^
1	14.171	−0.335	0.309	0.885
2	14.770	−0.278	0.276	0.906
3	13.606	−0.485	0.283	0.876
4	10.086	−0.377	0.270	0.844
5	11.819	−0.443	0.286	0.917
6	8.897	−0.441	0.305	0.783
7	13.618	−0.479	0.319	0.859
8	14.114	−0.450	0.291	0.847
9	13.763	−0.475	0.322	0.838
10	12.814	−0.398	0.267	0.866
11	13.636	−0.489	0.333	0.879
12	8.799	−0.522	0.299	0.833

*θ*_H_ is constant hydrotime, *Ψ*_b(50)_ is base water potential for 50% of seeds to germinate, and *σ*_φb_ is standard deviation of *Ψ*_b(50)_. The coefficient of determination (*r*^2^) shows the goodness of fit of the model. Three replications were performed for each treatment of each seed lot, and the above hydrotime model parameters were calculated using the average value of three replications of germination data.

**Table 3:** Effects of different environmental conditions (control, water stress, salinity stress, deep sowing, and cold stress) on seedling emergence percentage, seedling dry weight, and simplified vigor index of 12 seed lots of Chinese milk vetch (*Astragalus sinicus* L.) in pot experiments.

Seed Lot	Control Conditions			Water Stress			Salinity Stress			Deep Sowing			Cold Stress		
	PEP (%)	SDW (mg plant^−1^)	SVI	PEP (%)	SDW (mg plant^−1^)	SVI	PEP (%)	SDW (mg plant^−1^)	SVI	PEP (%)	SDW (mg plant^−1^)	SVI	PEP (%)	SDW (mg plant^−1^)	SVI
1	66.7 ab	49.9 abc	3280.1 de	35.6 cd	26.1 cd	924.5 def	38.9 bcd	28.4 efg	1088.3 cd	28.9 cd	15.3 b	432.3 de	41.1 cd	22.7 cdef	924.9 e
2	54.4 b	39.7 d	2153.4 f	28.9 d	15.4 f	433.5 g	26.7 d	24.1 g	631.2 e	15.6 e	9.0 c	135.2 f	32.2 e	17.7 f	561.1 f
3	77.8 a	53.9 ab	4175.9 ab	43.3 abc	34.6 ab	1484.9 bc	41.1 bc	30.9 def	1262.6 bcd	33.3 abcd	22.1 a	729.4 c	44.4 bc	25.0 bcde	1099.1 de
4	73.3 a	45.3 cd	3306.8 de	41.1 bc	20.7 e	849.4 f	44.4 abc	26.3 fg	1173.8 bcd	31.1 bcd	14.7 b	453.2 de	48.9 abc	22.0 def	1079.3 de
5	77.8 a	43.4 cd	3360.9 cde	45.6 abc	23.2 de	1045.5 de	43.3 abc	33.7 cde	1465.6 bc	32.2 bcd	12.9 b	408.3 de	50.0 abc	27.0 bcd	1350.1 bc
6	80.0 a	50.2 abc	4029.1 abc	44.4 abc	29.9 bc	1324.0 c	46.7 abc	27.3 fg	1262.0 bcd	38.9 abc	21.9 a	844.7 b	54.4 ab	22.0 def	1194.5 cd
7	74.4 a	56.9 a	4213.2 ab	47.8 ab	32.5 ab	1540.0 b	51.1 ab	40.8 ab	2093.2 a	40.0 ab	25.2 a	1008.3 a	53.3 ab	29.5 b	1538.2 b
8	72.2 ab	42.4 cd	3048.9 e	41.1 bc	26.4 cd	1081.2 d	42.2 abc	35.6 bcd	1497.3 b	32.2 bcd	16.1 b	508.7 d	52.2 abc	27.3 bcd	1425.7 bc
9	81.1 a	47.3 bcd	3846.3 bcd	37.8 bcd	23.1 de	869.3 ef	38.9 bcd	37.2 abc	1434.6 bc	26.7 d	13.1 b	346.5 e	43.3 bcd	21.7 def	927.8 e
10	66.7 ab	42.8 cd	2848.3 e	35.6 cd	23.9 de	848.0 f	34.4 cd	26.8 fg	919.6 de	25.6 d	16.2 b	396.8 de	43.3 bcd	20.4 ef	886.1 e
11	78.9 a	50.2 abc	3943.0 abcd	46.7 ab	32.9 ab	1533.1 b	48.9 ab	39.2 abc	1912.9 a	41.1 ab	24.7 a	1011.0 a	52.2 abc	28.3 bc	1471.9 b
12	83.3 a	55.6 a	4629.4 a	52.2 a	36.8 a	1906.0 a	53.3 a	42.2 a	2241.6 a	43.3 a	25.6 a	1108.4 a	57.8 a	39.0 a	2235.9 a
ANOVA															
SV		*df*		PEP			SDW			SVI					
*SL*		11		***			***			***					
*EC*		4		***			***			***					
*SL* × *EC*		44		ns			***			***					

*** Indicates significance at *p* < 0.001 probability level; ns indicates not significant at *p* < 0.05 probability level. Different lowercase letters within a column indicate significant difference at *p* < 0.05 probability level according to Duncan’s multiple comparison test. Emergence percentage data were arcsine-transformed before statistical analysis, and non-transformed data are shown in the table. Three replications were performed for each treatment and each seed lot, and each point is the average value of three replications. PEP: pot emergence percentage; SDW: seedling dry weight; SVI: simplified vigor index; ANOVA: analysis of variance; SV: source of variance; *df*: degrees of freedom; SL: seed lot; EC: environmental condition.

**Table 4:** Pearson correlation coefficient (*r*) between overall mean of germination percentage, germination rate, and germination index (overall mean of five levels of water potential and three replications), emergence percentage, seedling dry weight, and simplified vigor index under different environmental conditions (control, water stress, salinity stress, deep sowing, and cold stress) and in two field sowings with hydrotime model parameters (*θ*_H_, *Ψ*_b(50)_, and *σ*_φb_) for 12 Chinese milk vetch (*Astragalus sinicus* L.) seed lots.

Experiment Conditions	Variable	*θ*_H_ (MPa·h)	*Ψ*_b(50)_ (MPa)	*σ*_φb_
Germination test	Germination percentage (%)	−0.463	−0.972 (<0.001)	0.424
	Germination rate (day^−1^)	−0.702 (0.011)	−0.847 (0.001)	0.234
	Germination index	−0.623 (0.030)	−0.929 (<0.001)	0.312
Pot	Control conditions			
	Emergence percentage (%)	−0.531	−0.900 (<0.001)	0.476
	Seedling dry weight (mg plant^−1^)	−0.268	−0.642 (0.024)	0.546
	Simplified vigor index	−0.458	−0.858 (<0.001)	0.563
	Water stress			
	Emergence percentage (%)	−0.557	−0.853 (<0.001)	0.391
	Seedling dry weight (mg plant^−1^)	−0.290	−0.807 (0.002)	0.476
	Simplified vigor index	−0.415	−0.845 (0.001)	0.462
	Salinity stress			
	Emergence percentage (%)	−0.552	−0.780 (0.003)	0.504
	Seedling dry weight (mg plant^−1^)	−0.031	−0.820 (0.001)	0.666 (0.018)
	Simplified vigor index	−0.284	−0.834 (0.001)	0.616 (0.033)
	Deep sowing			
	Emergence percentage (%)	−0.523	−0.803 (0.002)	0.518
	Seedling dry weight (mg plant^−1^)	−0.347	−0.742 (0.006)	0.485
	Simplified vigor index	−0.423	−0.754 (0.005)	0.529
	Cold stress			
	Emergence percentage (%)	−0.630 (0.028)	−0.776 (0.003)	0.351
	Seedling dry weight (mg plant^−1^)	−0.375	−0.734 (0.007)	0.341
	Simplified vigor index	−0.508	−0.763 (0.004)	0.335
Field	First sowing			
	Emergence percentage (%)	−0.567	−0.825 (0.001)	0.519
	Seedling dry weight (mg plant^−1^)	−0.191	−0.600 (0.039)	0.527
	Simplified vigor index	−0.469	−0.810 (0.001)	0.566
	Second sowing			
	Emergence percentage (%)	−0.437	−0.884 (<0.001)	0.397
	Seedling dry weight (mg plant^−1^)	−0.383	−0.741 (0.006)	0.700 (0.011)
	Simplified vigor index	−0.490	−0.883 (<0.001)	0.617 (0.033)

*θ*_H_ is constant hydrotime, *Ψ*_b(50)_ is base water potential for 50% of seeds to germinate, and *σ*_φb_ is standard deviation of *Ψ*_b(50)_. Numbers in brackets indicate the significance of the coefficients. Three replications were performed for each treatment of each seed lot, and the correlation analysis was performed using the average value of three replications of emergence data.

**Table 5:** Production year, storage period, initial seed moisture content (SMC), thousand-seed weight (TSW), and proportion of hard seeds (HS) in 12 seed lots of Chinese milk vetch (*Astragalus sinicus* L.).

Seed Lot	Production Year	Storage Period (Years)	SMC (%)	TSW (g)	Proportion of HS (%)
1	2018	4	8.76	3.359	2.2
2	2016	6	8.52	3.392	2.2
3	2017	5	8.69	3.383	0.0
4	2017	5	8.61	3.389	1.1
5	2019	3	8.69	3.501	2.2
6	2018	4	8.48	3.384	1.1
7	2018	4	8.57	3.462	4.4
8	2019	3	8.86	3.431	2.2
9	2019	3	8.88	3.480	2.2
10	2017	5	8.72	3.427	0.0
11	2021	1	9.10	3.390	3.3
12	2021	1	8.93	3.415	2.2

## Data Availability

The data presented in this study are available on request from the corresponding author.
